# Evaluation of the efficacy of bleach routinely used in health facilities against *Mycobacterium tuberculosis* isolates in Ethiopia

**DOI:** 10.11604/pamj.2015.21.317.5456

**Published:** 2015-08-28

**Authors:** Daniel Mekonnen, Aschalew Admassu, Belaynew Wassie, Fantahun Biadglegne

**Affiliations:** 1 Bahir Dar University, College of Medicine and Health Sciences, Department of Medical Microbiology, Immunology and Parasitology, Bahir Dar, Ethiopia; 2 Bahir Dar Regional Health Research Laboratory Center, Department of Regional Mycobacteriology Laboratory, Bahir Dar, Ethiopia; 3 Bahir Dar University, College of Medicine and Health Sciences, School of Public Health; 4 Institute of Medical Microbiology and Epidemiology of Infectious Diseases, Medical Faculty, University of Leipzig, Germany

**Keywords:** Efficacy, Bleach, M. tuberculosis, Ethiopia, organic load

## Abstract

**Introduction:**

In Ethiopia, the most widely used disinfectant is 5% Hypochlorites. However, Ethiopian national health safety and infection prevention guideline recommendation on the use of bleach is not consistent and varying from 0.1%-4%. The purpose of this study was therefore to assess the effective time-concentration relationship of sodium hypochlorite against Mycobacterium tuberculosis complex isolates in the absence of any organic load.

**Methods:**

This experimental study was conducted in Bahir Dar Regional laboratory from February-June 2013. Test suspensions of 1.5 X 10^8^ CFU/ml prepared using normal saline containing 0.5% tween 80. From 5% stock, 0.1%, 0.5%, 1% and 2% bleach was prepared. A 1ml of test strain suspension and 1ml of bleach mixed and allowed to stand until the specified time achieved, neutralized by 48 ml phosphate buffer. 100µl from the diluted sediment were spread on two L-J mediums and incubated at 37°C for 8 weeks.

**Results:**

When 0. 1% bleach was used for 10 min, majority 11/20 of isolates showed 3 x 10^3^ CFU/ml growth (ME = 4.4) which was inefficient. However, when the time increased, the log10 reduction was acceptable, ME >5 and it was effective. The bleach solution containing 0.5% and above was effective in all respective times. In this study, there is no difference observed in the tuberculocidal activity of bleach against resistant and sensitive strains.

**Conclusion:**

Our study showed that in the absence of any organic load, 0.1% bleaches over 15 min and 0.5% bleaches over 10 min was found to be tuberculocidal.

## Introduction

Antiseptics and disinfectants are extensively used in health care settings for a variety of purposes. A wide variety of active chemical agents (“biocides”) including phenols, aldehydes, biguanides, surface-active agents, halogens, alcohols, iodine and others have been used for hundreds of years [1, 2]. Most of these active agents demonstrate broad-spectrum antimicrobial activity. However, little is known about the mode of action of these agents at different concentrations. Biocidal molecules and their formulations target multiple sites of the bacterial cell and mode of action depends on the physicochemical nature of the given molecule [1, 2]. Some biocides act as membrane destabilizers, and others are alkylating or oxidizing agents or intercalate with nucleic acids [2]. The widespread use of antiseptic and disinfectant products has prompted some assumption on the progress of microbial resistance; in particular cross-resistance to antibiotics. Antimicrobial activity can be influenced by many factors such as formulation effects, presence of an organic load, synergy, temperature, dilution, and test method [2–4]. Hypochlorites, the most widely utilized chlorine disinfectants, are available as liquid (sodium hypochlorite) or solid (calcium hypochlorite) [3]. The most commonly used chloro products in Ethiopian health care facilities are aqueous solutions of 5% sodium hypochlorite usually called household bleach (Chora Gas and chemical producing Factory, Addis Ababa, Ethiopia). However, its effective time-concentration against Mycobacterium tuberculosis complex (MTBC) and other vegetative bacteria is not well known. Ethiopian national health safety and infection prevention guideline recommendation on the use of bleach as a disinfectant is not consistent and varying from 0.1%-4% [5, 6].

Different studies indicated thatMycobacteria are well known for their resistance to disinfectants [1, 7, 8]. Their unusual high cell wall lipid content and the resultant hydrophobicity contributed to this resistance [1, 9]. Lack of proper and standardized test protocols has rendered the data on the tuberculocidal efficacy of chemical disinfectants unreliable and variable efficacy data [7, 9]. Moreover, the knowledge, attitude and practice of health care workers towards use of bleach in health care setting vary widely and inconsistent (unpublished data). This would have negative implication in both sides. Proper dilution of concentrated is important to get the full benefit of the germicide. By using a dilution higher that what is recommended, either by intention or by accident, has no any additional benefit. It will only be more expensive as you will purchase more chemical [10]. In Ethiopia, there is lack of information on the existing use of sodium hypochlorite against MTBC. Moreover, when we go through detail description of the bleach used in our country, it does not claim any time- concentration and it has no any material safety data sheet. The purpose of this study was therefore to assess the effective time-concentration relationship of sodium hypochlorite (NaOCl) disinfectant against MTBC isolates in the absence of any organic load.

## Methods

### Study Design and setting

This experimental quantitative suspension test study was conducted in Bahir Dar Regional Health Research Laboratory Center (BRHRLC) from February-June 2013. In quantitative methods, the number of surviving organisms is counted and compared to the original inoculum size. By subtracting the logarithm of the former from the logarithm of the latter, the decimal log reduction or microbicidal effect (ME) was obtained. A ME of 1 equals to a killing of 90% of the initial number of bacteria, ME of 2 means 99% killed. A generally accepted requirement was a ME that equals or was greater than 5: at least 99.999% of the germs were killed.

### Preparation of test strain suspension and working bleach

Specimens were collected using 50ml falcon tubes from all zones of ANRS, transported to BRHRLC based on WHO recommendation; Biological substance category B, UN-3337 standards. The collected specimens processed and decontaminated by the conventional N-acetyl-L-cysteine -NaOH (NALC-NaOH) method. After decontamination, the concentrated sediment was re-suspended in 1.0 ml sterile phosphate buffer (PBS, pH =6.8). From this, 100 µl of sediment was inoculated on two Lowenstein Jensen (LJ) mediums and incubated at 37^0^C for maximum of 8 weeks. Test strain suspensions were prepared by suspending harvested MTBC grown on Lowenstein Jensen (LJ) media in sterile normal saline containing 0.5% Tween 80 and homogenizing them for 1 min with sterile glass beads to obtain 1.5 X 10^8^ CFU/ml. The concentration of available chlorine in bleach can be expressed in percentage, parts per million (ppm) and g/l. The currently used stock solution of bleach (called sedex berekina in local Amharic language) contains 5% chlorine. From this 5% stock, we prepared 0.1%, 0.5%, 1% and 2% fresh working bleach using the following formula [11].

C1V1 = C2V2

Where V1 = the volume of stock solution required to prepare working solution, C1= the concentration of chlorine in the stock solution expressed in percentage, V2= the volume of newly prepared working solution from the stock and water, C2= the concentration of chlorine in the newly prepared working solution. For one patch we prepared 60ml of each of working solution (0.1%, 0.5%, 1%, 2% and 5%) as follows.

C1V1 = C2V2; 5% V1 = 0.1%60ml

60ml of 0.1% (1000ppm) bleach solution had been prepared by combining 1.2 ml of 5% beach with 58.8 ml of sterile distilled water. All the other working solution prepared in the same fashion. Moreover, our percentage working solution have been used by converting in to ppm using the formula below [10], PPm= (% of active ingredient x 10,000 dilution rate of product): 0.1% bleach = 0.1X10, 000 = 1000ppm; 0.5% beach = 0.5X10, 000 = 5000ppm; 1% bleach = 1X10, 000 = 10,000ppm; 2% bleach = 2X10, 000 = 20,000ppm; 5%bleach = 5 X10, 000 = 50,000ppm.

### Laboratory procedures

**Figure 1:**
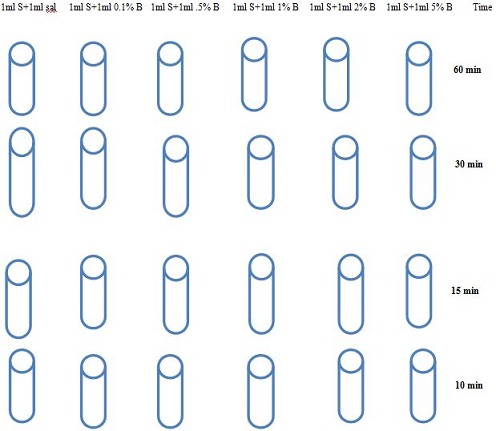
Concentration-time arrangements for experiments on tuberculocidal activity of bleach against 0.5MacFarland (1.5x10^8^CFU/ml) MTBC isolates, Bahir Dar, Ethiopia, 2013

One ml each of (0.1%,0.5%,1%,2%and 5%) freshly prepared bleach was added in to 5 test tubes of 50 ml capacity. The remaining bleach was used for pH and temperature measurement. From 1 ml of bleach, 1 ml of 1.5 X 10^8^ CFU/ml of test strain was added, mixed by vortex and allowed to stand until specified time achieved (Figure 1). Phosphate buffer solution of 850ml (PBS, pH = 6.8) prepared in blue cap bottle of 1000 ml size. After the required contact time, 48 ml PBS as neutralizer added and centrifuged at 10000g for 15minute in safety centrifuge which has 4^0^C to avoid the heat lethal effect. Supernatant discarded and then 2ml PBS as diluents was added. Samples (100µl) from the diluted sediment were spread on two L-J agar medium and incubated at 37°C for 8 weeks. Controls for each suspension contained 2 ml of equal volumes of sterile normal saline and strain suspension making the final concentration of 7.5x10^7^CFU/ ml prepared. From this control, 100µl inoculated on two L-J agar medium and incubated the same way as test strain suspension. Growth colony graded, and the grade translated in to number of colonies. CFU/ml from control-CFU/ml from disinfectant added growth gave log10 reduction or ME. Effective concentration and time considered when the capacities to cause up to a 5-log (99.999%) reduction in CFU/ml of MTBC or ≥5 ME.

### Data Collection Procedures

The data were captured using data collection forms. It comprised of the Strain ID, room temperature, temperature, PH, and dilution (0.1% to 5%) of bleach, and the growth result of test organism in culture with respect to their exposure time in minutes (Table 1).

**Table 1:** Data collection sheet for the experimental study on efficacy of bleach against MTBC isolates, Bahir Dar, Ethiopia, 2013

Strain	Room T^0^	Bleach T^0^	pH of Bleach	Concentration of Bleach (%)	Growth of *M. tuberculosis* on LJ after exposure for:			
					10 min	15min	30 min	60 min
				0.0				
				0.1				
				0.5				
				1.0				
				2.0				
				5.0				

T^0^= Temperature

### Data Quality Assurance

Neutralizers used in this study have been verified in accordance with Ethiopian tuberculosis culture laboratory neutralization practice during sputum processing and 48 ml of PBS (PH = 6.8) was used. The sterility of reagents and media, and the performance of the LJ media have been verified. Moreover, the temperature of the room and the incubator was monitored daily using calibrated thermometer. Furthermore, the pH and temperature of bleach was measured using microprocessor based pH bench meter (*HANNA instruments inc.USA*).

### Definitions

**Antiseptics:** chemical agents that inhibit or kill microbial growth and are nontoxic when applied to living tissues, used for hand washing or for treating surface wounds. Under certain circumstances, some antiseptics are also effective disinfectants.

**Disinfectants:** chemical and/or physical agents used to destroy or irreversibly inactivate many or all of the pathogenic microorganisms but not necessarily spores and not all viruses.

**Free chlorine:** combined forms of HOCl (hypochlorous acid), OCl^-^ (hypochlorite anion) and Cl2 (dissolved chlorine gas) in aqueous solution.

**Combined chlorine:** chlorine in water in chemical combination with ammonia to form inorganic amines, or with organic compounds to form organic amines.

**Total chlorine:** the sum of free and combined chlorine. For clean water, the total chlorine is essentially equal to free chlorine. When chloramines are present the total chlorine will be higher than the free chlorine.

**Efficacy:** is the ability of bleach to completely destroyed MTBC.

**Biocide:** a chemical agent that inactivates microorganisms.

**Synonym:** Chlorine Bleach, Bleach, Soda Bleach, Chlorox; Sodium Hypochlorite, 5% Available Chlorine.

### Ethical Considerations

Ethical clearance and permission obtained from the Department ethics committee of Amhara Regional State Health Bureau and letter of support and clearance obtained from research and technology transfer core process.

## Results

In this study, 10 multidrug resistant and 10 sensitive MTBC strains were used for assessing the tuberculocidal effect of bleach with specified concentration and exposure time. We used 24 test tubes for a single isolate to determine the efficacy of bleach at different time intervals. Data collection sheet were used to collect information on important variables i.e. pH and temperature of bleach; pH and temperature of water used and temperature of the room (Table 2, Table 3). The mean pH and temperature of the 5% (50,000ppm) bleach was 13.07 and 23.4 ^0^C which was higher compared to other lower working solutions. In this study, we found that the pH of bleach decreased as concentration of bleach decreased (Table 2).

**Table 2:** pH of each specific concentration of bleach used in the experiment for assessing the effective time-concentration of bleach against MTBC isolates, Bahir Dar, Ethiopia, 2013

	pH of bleach at Concentration of:				
	0.1%	0.5%	1%	2%	5%
**Mean**	11.7152	12.2848	12.5233	12.7657	13.0738
**Median**	11.7400	12.2700	12.5300	12.7700	13.0900
**SD**	0.15197	0.07756	0.09671	0.06038	0.03008
**Minimum**	11.54	12.16	12.32	12.61	13.01
**Maximum**	11.96	12.42	12.67	12.85	13.10

**Table 3:** Temperature of each specific concentration of bleach used in the experiment for assessing the effective time-concentration of bleach against MTBC isolates, Bahir Dar, Ethiopia, 2013

	Temperature of bleach at Concentration of:				
	0.1%	0.5%	1%	2%	5%
**Mean**	22.4714	22.2762	22.4524	22.7952	23.3714
**Median**	23.7000	23.6000	23.6000	23.6000	23.4000
**SD**	2.39753	2.79963	2.61699	2.02101	1.00705
**Minimum**	17.80	16.80	17.40	18.70	22.00
**Maximum**	24.10	24.40	24.30	24.30	24.50

### Growth rate of isolates after exposure under different bleach concentrations

When 1ml of 0.5 MacFarland of isolates was mixed with 1ml of 0.1% dilution of bleach for 10 min, majority 11/20 (55%) of isolates showed 3 x 10^3^ CFU/ml growth (ME = 4.4), 6/20 (30%) showed 5 x 10^3^ CFU/ml growth (ME = 4.2) and the rest 3/20 (15%) showed 57 CFU/ml growth (ME = 5.9). This showed that 0.1% bleach for 10min exposure was ineffective. It was unable to produce more than a 5-log10 (≥5 ME) reduction in all tubes. However, when the time increased (15min, 20 min, 30min and 60 min), the log10 reduction was acceptable, ME >5 and it was effective. The sodium hypochlorite solution containing above 5,000 ppm (0.5%) of available chlorine was effective in all respective times, producing above 5-log10 reduction (Table 4). Moreover, in all tests, control reactions containing no disinfectant resulted in complete recovery of the initial inoculums, 7.5x10^7^ CFU/ml growth.

**Table 4:** Killing Log value of bleach after exposure of isolates for specified time-concentration, Bahir Dar, Ehiopia, 2013

Concentrationof bleach (%):	log10 reduction in CFU/ml after exposure time of:				
	10 min	15 min	20min	30min	60 min
**0.1**	4.4	>5	>7.9	>7.9	>7.9
**0.5**	>7.9	>7.9	>7.9	>7.9	>7.9
**1**	>7.9	>7.9	>7.9	>7.9	>7.9
**2**	>7.9	>7.9	>7.9	>7.9	>7.9
**5**	>7.9	>7.9	>7.9	>7.9	>7.9

## Discussion

We confirmed that the pH and efficacy or log reduction ability increased as the concentration of chlorine increased. This seems contrary to what literatures claimed on pH and efficacy of bleach. When the pH is between 2-7, the equilibrium favors HOCl. As the pH falls below 2, the main form is Cl2. At a pH of 7.4, HOCl and OCl^-^ are about equal, and as the pH goes above 7.4, increasing proportions of OCl^-^ are present [12]. Maximum disinfecting efficacy is achieved at pH 4-5, because essentially all the chlorine is present as HOCl which is two orders of magnitude more effective than OCl^-^ [3, 7, 12]. Chlorine gas is quite toxic, so pH below 4 should be avoided. On balance, for safety and efficacy a pH of 5-7 works best [12]. Alkaline germicides have pH of 8-14. The higher the pH, the better cleaning, and decreasing properties a germicide will have. However, cleaner/germicides over pH 10 can be harmful to floor finishes [10]. Another literature claims similar pH range to us. Commercial household liquid bleach ranges from 5 - 10% sodium hypochlorite and has a pH of 11 to 12. Sodium hypochlorite is toxic due to the hypochlorite moiety that is formed when sodium hypochlorite is dissolved in water in alkaline conditions [13].

Studies conducted in different countries revealed different results on the efficacy of bleach against tuberculosis. Higher concentrations, 1% (10,000 ppm) of chlorine are required to kill *M. tuberculosis* in 1minute [9]. However, smaller chlorine concentrations such as 0.0006-0.01% found to be less effective on Mycobacterium. Moreover, the efficacy of sodium hypochlorite was slightly reduced in the presence of sputum [14]. Its concentration also varies according to the time and the specimens used [6]. *M. tuberculosis* in sputum was successfully sterilized by adding equal volumes of 15% bleach for one minute, 6% for five minutes or 3% for 20 minutes [15]. Studies revealed that the concentration of bleach and time applied to decontaminate biohazard spill varied from 0.05%-0.5% for 15-20 min [16, 17]. Likewise, heavy contamination and BSC could be contaminated at 0.2% and 1% for 15 minute [17]. A study by Ascenzi JM et al in 1987 aimed to determine a more accurate method (1:10 volume of strain suspension and disinfectant) for measurement of tuberculocidal activity of disinfectants. The recovery of *M. bovis* after 2min and 5 min exposure by 0.05% bleach was 43 and 0 CFU/ml respectively indicating that increasing time at equal concentration of hypochlorite is found to be tuberculocidal. However, the results obtained in this organism cannot be considered relevant when dealing with slow-growing Mycobacteria [18]. Because of differences in methodologies, type of disinfectant product and difference in standards of time for claiming efficacy, it is difficult to compare our study with other studies. In Ethiopia, the most recommended time frame is 10 min, 15 min, 20 min, 30 min and 60 min [6]. Others potential reasons for discrepancy was due to difference in preparation of the ratio of test suspension and disinfectant, in our case equal proportion of test inoculums and disinfectant was used, which is a common practice in tuberculosis laboratory for decontamination of liquid wastes like sputum. The other difference was the type of isolates used, for this study we used MTBC isolates but others used non infectious *M. tuberculosis* (H37Rv) and *M. bovis*. In this study, there is no difference observed in the tuberculocidal activity of bleach against resistant and sensitive MTBC strains. Bacterial resistant to antibacterial agents is either a natural property of an organism (intrinsic) or acquired. However, to date, plasmid or transposon-mediated resistance to biocides has not been demonstrated in mycobacteria [7]. The use of disinfectant in health facility is mandatory to prevent nosocomial infection. The spread of tuberculosis through the use of improperly disinfected bronchoscopes and endoscope has been documented [1, 9]. A total of 80% of laboratory-acquired tuberculosis cases have resulted from no obvious cause and it has been suggested that the use of ineffective chemical disinfectants may be responsible for some of these cases of laboratory acquired infections [9].

## Conclusion

The study showed that 0.1% bleach over 15min and 0.5% bleach over 10 min was tuberculocidal in the absence of any organic load. However, decontamination of tuberculosis containing sample required higher concentration of bleach (i.e. 0.5 for 15 min, 1% for 10min). Moreover, this study confirmed that there was no difference in the bleach, disinfectant in susceptibility of sensitive and resistance MTBC stains. Currently, Ethiopian National and Regional Mycobcteriology laboratories are using 2% bleach for 60 min for decontamination of leftover sputum samples before taking it for incineration. According to this finding, this practice is over use of bleach. It is advisable to use the correct and appropriate concentration of bleach. There is no any additional benefit using dilution that is higher than what is recommended. It will only be more expensive as you will be purchasing more chemical. Moreover, using high concentrated bleach result in health risks. Infection Control Committees in health care facilities, made up of doctors, nurses and/or decision makers are responsible deciding and approving the effective concentration-time of bleach, disinfectants and cleaning methods to be employed for each component of their medical facilities.
